# miR-24, miR-30b and miR-142-3p interfere with antigen processing and presentation by primary macrophages and dendritic cells

**DOI:** 10.1038/srep32925

**Published:** 2016-09-09

**Authors:** Afsar Raza Naqvi, Jezrom B. Fordham, Balaji Ganesh, Salvador Nares

**Affiliations:** 1 Department of Periodontology, University of Illinois at Chicago, Chicago, Illinois, USA; 2 Research Resources Center, University of Illinois at Chicago, Chicago, Illinois, USA

## Abstract

Antigen uptake, processing and presentation by antigen presenting cells (APCs) are tightly coupled processes which consequently lead to the activation of innate and adaptive immune responses. However, the regulatory role of microRNA (miRNAs) in these critical pathways is poorly understood. In this study, we show that overexpression of miR-24, miR-30b and miR-142-3p attenuates uptake and processing of soluble antigen ovalbumin (Ova) in primary human macrophages and dendritic cells. MiRNA mimic transfected APCs exhibit defects in antigen presentation (Ova and CMV antigen) to CD4+ T-cells leading to reduced cell proliferation. Using transgenic OT-II mice we demonstrated that this impairment in T-cell proliferation is specific to antigen provided i.e., Ova. Further, human T-cells co-cultured with miRNA transfected dendritic cells secrete low levels of T helper (Th)-1 polarization associated cytokines. Analysis of molecules regulating APC and T-cell receptor interaction shows miRNA-mediated induced expression of Programmed Death-Ligand 1 (PD-L1) which inhibits T-cell proliferation. Blocking PD-L1 with antibodies rescues miRNA-mediated inhibition of T cell priming by DCs. These results uncover regulatory functions of miR-24, miR-30b and miR-142-3p in pairing innate and adaptive components of immunity.

Macrophages (MΦ) and dendritic cells (DC) are antigen presenting cells (APCs) strategically poised along portals of entry where they perform functions of vital importance to host survival. These cells are active participants in innate immunity and orchestrate the transition to- and propagation of- the adaptive arm of the immune response^1^,^2^. Critical for their function is their ability to internalize particles and process antigen for subsequent presentation to T-cells^2^,^3^. Depending on the nature of antigen, there are four different pathways for the internalization of exogenous antigens by APCs *viz*., endocytosis, pinocytosis, phagocytosis and macropinocytosis^4^,^5^. In general, internalized particles are trafficked into the progressively acidic endosomal network where they are degraded into smaller peptides and subsequently loaded onto major histocompatibility complex (MHC)-II molecules for presentation. Receptors involved in the recognition of particles favors efficient uptake and control trafficking to the target compartment thereby increasing their proper processing and presentation^6^,^7^. Because of their impact on host defense, these pathways are tightly regulated at multiple levels. In this regard, the regulatory role of miRNAs is increasingly recognized.

In metazoans, miRNAs represent a class of small (~21 nts), non-protein coding, RNA molecules that bind target mRNAs leading to their degradation or translational suppression^8^,^9^. They have been attributed with various functions including cell development, apoptosis, and signal transduction^8^,^10^. Recent work also demonstrates their key role in shaping of both innate and adaptive immunity^11^,^12^. For instance, miR-146a expression is induced upon LPS stimulation and target TLR signaling components leading to immune tolerance^13^. MiR-181 family members have been reported to modulate T-cell differentiation by downregulating expression of genes (Bcl-2, CD69, TCR) that are involved in positive and negative T-cell selection while, miR-21 targets IL-2 and IFN-γ signaling processes that regulate Th1 and Th2 polarization and inflammatory responses^14^,^15^,^16^,^17^,^18^.

Productive antigen presentation commences with particle internalization followed by processing and culminates with antigen presentation to cells of the adaptive arm of immunity, *viz*., T- or B- cells. However, the role of miRNAs in this regard remains largely unknown. Identifying miRNAs that regulate antigen processing and presentation is critical to our understanding of this key leukocyte function. Previously, we demonstrated that ectopic expression of miR-24, miR-30b and miR-142-3p, differentially downregulated during monocyte to MΦ and DC development, attenuate uptake of bacteria in myeloid inflammatory cells^19^,^20^. In this study, we examined the role of miR-24, miR-30b and miR-142-3p in the uptake and processing of soluble antigen (Ovalbumin). Time kinetics analysis reveals differential defects in miRNA mimic transfected MΦ and DC. We further demonstrated the inhibitory impact of these candidate miRNAs on antigen presentation to T-cells by MΦ and DC. This corroborates with attenuated T-cell proliferation as well as cytokine production. We examined and identified miRNA-mediated changes in surface expression of stimulatory and inhibitory molecules present on MΦ and DC. These findings highlight the crucial role of miRNAs in regulating the immune response and their immense therapeutic potential in regulating critical functions of myeloid-derived inflammatory cells.

## Results

### MΦ and DC overexpressing miR-24, miR-30b, and miR-142-3p exhibit impaired antigen processing

**Figure 1:**
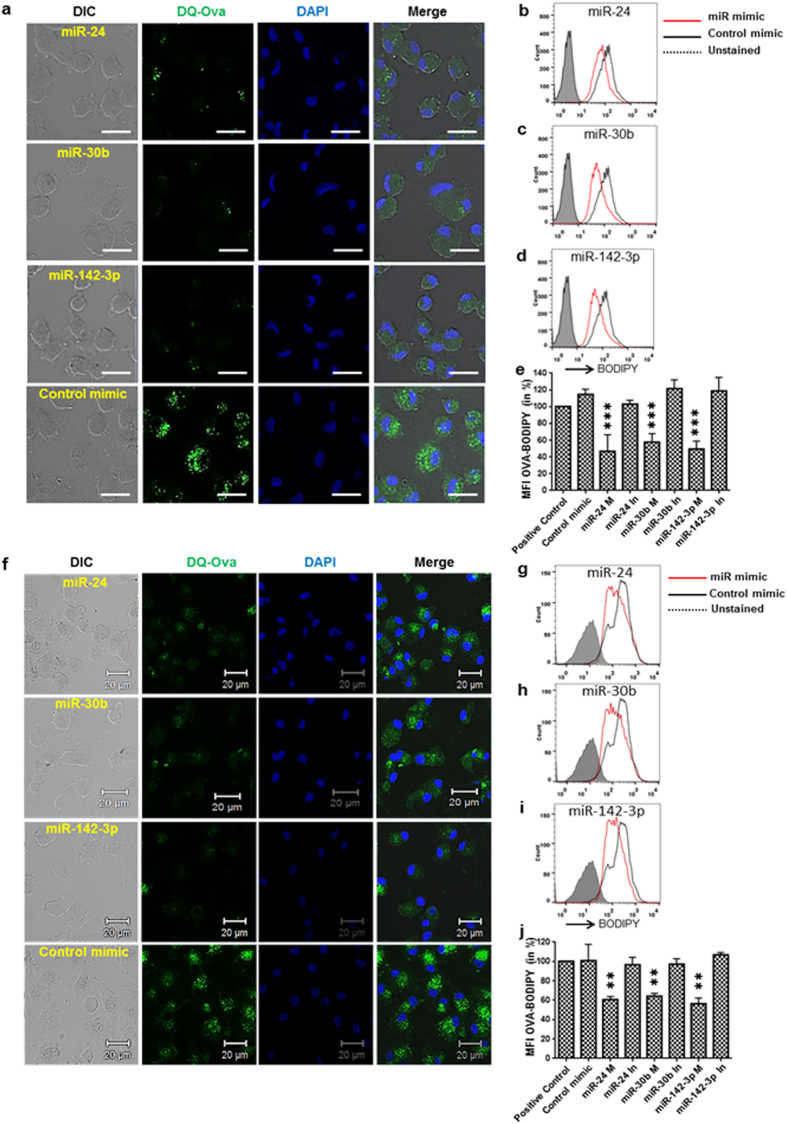
MΦ and DCs overexpressing miR-24, miR-30b, and miR-142-3p are defective in antigen processing. (**a**) MΦ and (**f**) DC were transfected with miRNA or control mimics and incubated with DQ-OVA for 2 hours. Representative confocal images showing inhibitory impact of miRNAs on antigen processing by APCs. Green signals indicate processing of DQ-OVA which otherwise is quenched, Scale bar, 20 μm. MΦ and DC were harvested after 2 hours of DQ-OVA incubation and analyzed by flow cytometry. Overlay of histograms of miRNA or control mimic transfected cells showing differences in BODIPY dye signals in (**b**–**d**) MΦ and (**g**–**i**) DC which reflect antigen processing capability of the cells. Histograms showing percent geo. MFI values in miRNA mimics (M), inhibitor (In) or control mimic transfected (**e**) MΦ and (**j**) DC. The data presented as ±SEM of four independent experiments in each cell type. Student’s *t*-test was conducted to calculate p values. **P < 0.01, ***P < 0.001.

We have recently shown that down-regulation of miR-24, -30b and -142-3p during MΦ and DC differentiation is necessary for acquisition of the functional phenotype^19^. The impact of enforced expression of these miRNAs was examined on the phagocytosis of bacteria and opsonized beads by MΦ and DC where we demonstrated that uptake by two different receptor mediated pathways is attenuated by miRNAs (Naqvi A.R., *In press*)^20^,^21^. Uptake of antigen by APCs culminates with the processing of internalized particles for subsequent presentation by MHC molecules to adaptive immune cells. We therefore examined the impact of miR-24, miR-30b and miR-142-3p on antigen processing by MΦ and DC. As an antigen we used BODIPY FL dye (DQ) conjugated Ova, a soluble antigen. DQ-Ova is a self-quenched conjugate of Ova that exhibits bright green fluorescence upon proteolytic degradation. MΦ transfected with miR-24, miR-30b and miR-142-3p mimics show reduced green signal compared to control mimics (Fig. 1a) suggesting impaired antigen processing upon enforced expression of the miRNA mimics. To quantitate the defect in antigen processing, we harvested cells and examined fluorescence by flow cytometry. Overlay of histograms of control and miRNA mimics show marked differences in antigen processing (Fig. 1b–d). As a positive control, mock transfected cells were assayed for their capability to process Ova-BODIPY. We observed ~27%, 30% and 38% decrease in DQ-Ova degradation in MΦ transfected with miR-24, miR-30b or miR-142-3p, respectively, compared to control mimic (Fig. 1e). We then examined antigen processing in miRNA inhibitor transfected cells. Compared to control mimic, no significant differences were noted in the presence of miR-24, miR-30b or miR-142-3p inhibitor (Fig. 1e).

Next, we assessed if these miRNAs can also interfere with the antigen processing capability of DCs. Compared to control mimics, less processing of DQ-Ova was noticed in miRNA transfected DCs as reflected by low green BODIPY signal in confocal imaging (Fig. 1f). Flow cytometric analysis showed antigen processing was reduced to approximately 22%, 38% and 40% in DC overexpressing miR-24, miR-30b and miR-142-3p, respectively (Fig. 1g–j). Compared to control mimic, miRNA inhibitor had no significant effect on antigen processing (Fig. 1j). Therefore, subsequent experiments were performed with the focus on miRNA mimics. Together, these observations clearly show an inhibitory effect of miRNA overexpression on antigen processing by APCs.

### Time kinetics of antigen uptake and processing in MΦ and DC overexpressing miR-24, miR-30b, and miR-142-3p

**Figure 2:**
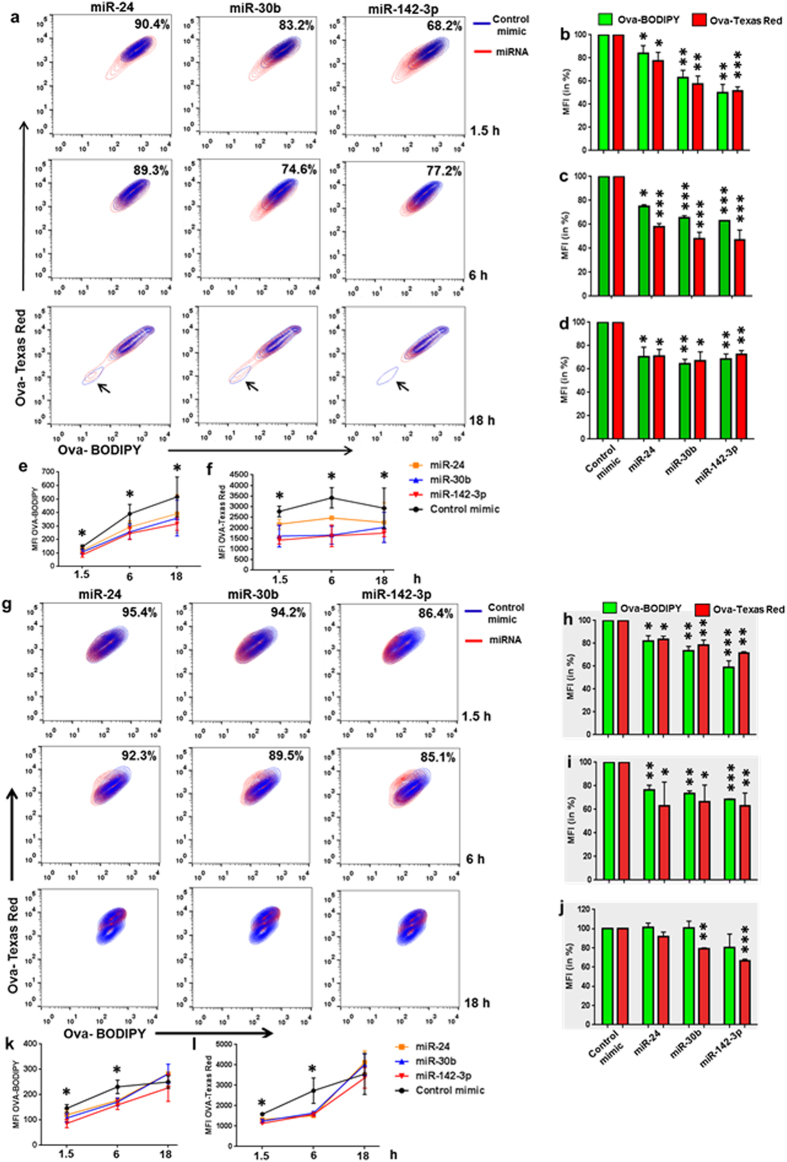
Time kinetics of antigen uptake and processing in miR-24, miR-30b, and miR-142-3p overexpressing APCs. Time kinetics of Ova uptake and processing was assessed using Texas Red and BODIPY (DQ) conjugated Ova, respectively. MiRNA mimic or control transfected (**a**) MΦ and (**g**) DC were incubated with Texas Red-Ova and DQ-Ova and the cells were harvested after 1.5, 6 and 18 hours. Contour plots showing overlay of miRNA and control mimic transfected cells positive for Texas Red and DQ and the percent overlap is mentioned in the right corner. Histograms showing geometric MFI values (in %) of (**b**–**d**) MΦ and (**h**–**j**) DC at different time points post Ova treatment. Data are also presented as MFI values for both conjugated Ova in (**e,f**) MΦ and (**k,l**) DC. The data are representative of three independent experiments (n = 4). *P < 0.05, **P < 0.01, ***P < 0.001 (two tailed Student’s *t*-test).

We next questioned whether the observed effect on antigen processing is a consequence of reduced Ova uptake. To test this, we assessed both antigen uptake and antigen processing concurrently. For this purpose, we used Texas red conjugated Ova which allowed us to monitor antigen uptake while, DQ-Ova would reflect antigen processing. We examined the time kinetics of these pathways by analyzing cells at three different time points: 1.5, 6 and 18 h. In MΦ, antigen uptake as well as antigen processing was markedly inhibited by miR-24, −30b and −142-3p across the time points examined (Fig. 2a). MiRNA transfected population (red) lags behind the control mimic (blue) as early as 1.5 h and was similarly impacted at 6 h. We gated the population of miRNA mimic transfected cells onto control mimic and noticed that a significant percent of cells lagged behind in ability to uptake and process antigen compared with control transfected cells (Fig. 2a; see percent overlap values on the upper right corner). MiRNA transfected cells, more specifically miR-142-3p, were also less efficient in clearing the antigen as reflected by the absence of population devoid of Ova (both Texas Red and BODIPY labelled Add; ‘see black arrows in Fig. 1, lower panels’.). Nonetheless, miRNA mimic transfected cells exhibited poor uptake and processing for up to 18 h as shown by reduced geometric MFI (mean fluorescence intensity) percentages and values (Fig. 2b–f).

Antigen uptake and processing by DCs was also negatively impacted by these miRNAs, however, they exhibit time responsive behavior with respect to antigen uptake and processing. Figure 2g shows that significant differences in miRNA and control mimic populations after 1.5 and 6 h. MFI values also show marked reduction in both antigen uptake and processing in miRNA transfected DCs (Fig. 2h,i,k,l). After 18 h post incubation, control cells show two clearly distinct populations implying Ova clearance, but this was not observed in miRNA transfected cells (Fig. 2g; lower panel). With regard to MFI, we noticed significant differences in the uptake of Ova in miR-30b and miR-142-3p transfected cells but not in miR-24 transfected cells compared to control mimic (Fig. 2j,l). On the other hand, antigen processing was not significantly different in miRNA or control mimic transfected DC at 18 h time point (Fig. 2j,k). However, it can be noted that even though there were less number of control mimic transfected cells undergoing active antigen uptake and processing at 18 h, the geo. MFI values were still similar, and not significantly reduced, compared to the miRNAs mimics (Fig. 2j; green bars). Overall, these results clearly show miR-24, −30b, and −142-3p mediated impairment of antigen uptake and processing in APCs.

### Impaired T-cell activation and proliferation by miR-24, miR-30b, and miR-142-3p transfected APCs

**Figure 3:**
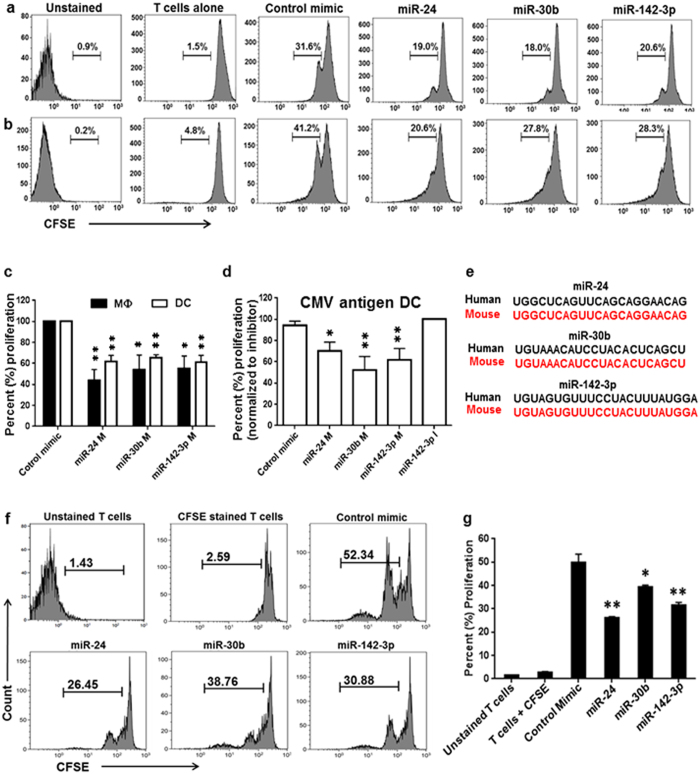
Impaired T-cell proliferation by MΦ and DC overexpressing miR-24, miR-30b and miR-142-3p. miRNA or control mimic transfected APCs were incubated with Ova (10 μg/ml). CFSE labeled autologous T-cells were co-cultured with APC at a 5:1 ratio and re-stimulated at day 3 with OVA (5 μg/ml). Day 10 co-cultured cells were harvested and analyzed by flow cytometry. Representative histograms showing proliferation of T cell co-cultured in presence of (**a**) MΦ and (**b**) DC as reflected by dilution of CFSE. CFSE unstained and stained T-cells were used as positive and negative controls. Values show percent proliferation after gating for CFSE stained and unstained T-cells. Images shown are representative of three independent donors. Experiments were performed at least thrice with similar results. (**c**) Percent T cell proliferation in miRNA or control transfected APCs co-cultured with T cells in presence of Ova. Proliferation is presented as control mimic normalized values (in %). (**d**) Same as in (**c**) except that CMV antigen was used instead of Ova and the T cell proliferation is presented as miR-142-3p inhibitor normalized values (in %). (**e**) Sequence alignment of human and mouse analogs of miR-24, −30b and −142-3p show 100% homology. (**f**) MiRNA-mediated antigen (Ova) specific proliferation defects in OT-II mice derived CD4+ T-cells. Day 7 cultured BMDCs were transfected with murine analogs of miR-24, −30b, −142-3p or control mimic. After 24 hours, cells were co-cultured with freshly spleen sorted and CFSE labeled CD4+ T-cells from OT-II mice. Cells were harvested after 4 days and analyzed by flow cytometry. Representative histograms showing Ova specific proliferation of T-cells double positive for CD4 and CFSE. (**g**) Percent T-cell proliferation in miRNA and control transfected cells. The data are representative of three separate experiments. *P < 0.05, **P < 0.01, ***P < 0.001 (two tailed Student’s *t*-test).

Productive antigen presentation requires efficient uptake of particles by APCs, most potent of which are DC and MΦ. APC present processed antigens to T-cells leading to their activation and subsequent proliferation. We investigated whether defective antigen processing is associated with inefficient T-cell proliferation by APC. To this end, APCs transfected with miR-24, miR-30b, miR-142-3p or control mimics were co-cultured with autologous carboxyfluorescein succinimidyl ester (CFSE) labeled CD4+ T-cells in presence of Ova and the rate of T cell proliferation was measured after 10 days. As evident from the data, higher T-cell proliferation was observed in T-cells co-cultured with DC than with MΦ. T-cells co-cultured with either MΦ or DC transfected with miRNA mimics, show significantly reduced proliferation (Fig. 3a,b). In MΦ, overexpression of miR-24, −30b or −142-3p reduced CD4+ T-cell proliferation by ~56%, 46%, 44%, respectively, compared to control mimic (Fig. 3c). On the other hand, DCs transfected with miR-24, −30b or −142-3p showed ~38%, 45% and 48% reduction in T-cell proliferation (Fig. 3c). These results show that impaired antigen uptake and processing corroborates with attenuated CD4+ T-cell proliferation.

To further validate that antigen mediated T cell priming by APC is compromised by miRNAs, we also used potent immunogenic CMV antigen instead of Ova which can induce T cell anergy. Compared with control, 30–50% reduced T cell proliferation was observed in miRNA mimics expressing DCs (Fig. 3d). To examine the specific impact of miRNA mimics, we also used miR-142-3p inhibitor. However, no significant difference between control mimic and inhibitor was observed. These results further indicate that miRNA-mediated antigen processing defects are associated with T cell proliferation.

### miR-24, miR-30b, and miR-142-3p impair Ova specific T-cell proliferation

Next we questioned if the defects in T-cell proliferation are specific to the antigen challenge. In order to evaluate Ova-specific T-cell proliferation by APCs, we performed antigen presentation assays using cells from T-cell receptor (TCR) transgenic mice (OT-II), which recognize Ova derived peptides. As miRNA-target interaction is sequence specific, we examined the sequence conservation of miR-24, −30b and −142-3p in human and mice analogs. Sequence alignment show 100% homology indicating common gene targets of these miRNAs (Fig. 3e). Similar to human, these miRNAs are also expressed in mice myeloid cells^22^,^23^. We therefore hypothesized that with conserved sequences and high expression in mice APCs, these miRNAs will functionally modulate mice APC activity. We first tested whether murine analogs of miR-24, −30b and −142-3p can impact antigen processing. Bone marrow derived DC (BMDC) were transfected with murine miR-24, −30b and −142-3p and functional assays performed. Comparable to human myeloid cells, we observed miRNA-mediated defects in antigen uptake and processing by BMDCs as observed by reduced Texas Red and BODIPY signals (Supplementary Figure 1a). We further quantified the Ova-BODIPY processing by flow cytometry analysis and noted ~30–50% reduction in antigen processing in miRNA transfected BMDC compared with control mimics (Supplementary Figure 1b). These results clearly show the conserved role of miRNAs in regulating antigen processing in both human and mice APCs. We next investigated the antigen presentation capability of OT-II mice derived BMDCs. CD4+ T-cells co-cultured with miR-24, −30b, or −142-3p transfected BMDCs respectively, show ~50%, 27% and 43% reduced proliferation compared with control mimic (Fig. 3f,g). These results support miRNA mediated inhibition of T-cell proliferation is specific to antigen.

### Overexpression of miR-24, miR-30b, and miR-142-3p suppress type I cytokines by DCs

**Figure 4:**
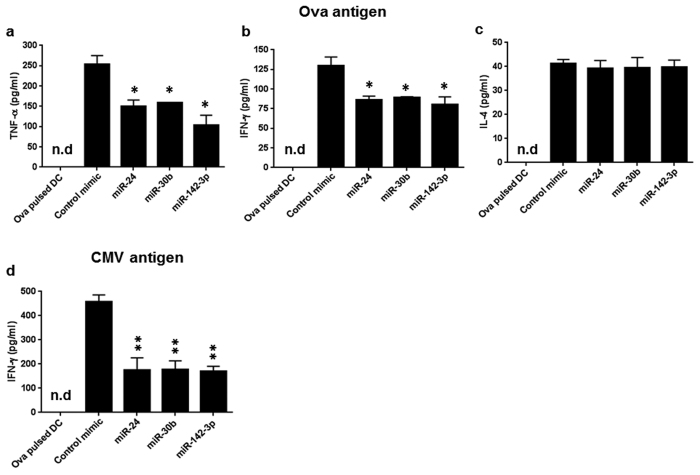
MiRNA overexpression alters secretion of Th1 cytokines in human DC and T-cell co-cultures. Supernatant was collected from co-cultures of human CD4 T-cells with miRNA mimic transfected DC primed with Ova or CMV antigen. Secreted levels of human cytokines (**a**) TNF-α, (**b**) IFN-γ, and (**c**) IL-4 were determined by ELISA in Ova challenged co-cultures. (**d**) IFN-γ levels in supernatant of DC-T cell co-cultures primed with CMV antigen. As a negative control, cytokines levels were examined in the supernatants of Ova pulsed DC cultured in the absence of T-cells. *P < 0.05, **P < 0.01 (two tailed Student’s *t*-test).

Upon activation by APCs, T-cells rapidly proliferate and secrete cytokines which facilitate different types of immune responses. Based on the cytokine profile, T-cells are categorized as Th1, Th2, Th3, Th17, Th9, or Tfh. To examine which Th population is impacted by overexpression of miRNA mimics in human DC, we examined various subset-associated cytokines, specifically Th1 (TNF-α and IFN-γ), Th2 (IL-4 and IL-10) and Th17 (IL-17) in the co-culture supernatants. Compared to control mimics, we noticed significantly lower TNF-α secretion in miR-24, miR-30b and miR-142-3p mimics (Fig. 4a). Similarly, Th1-associated IFN-γ, albeit detected at very low levels, was secreted to a lesser extent in miRNA mimic samples (Fig. 4b). However, CD3/CD28 treated T-cells that were used as a positive control show high expression of IFN-γ (data not shown). A low level of IL-4 (Th2 cytokine) was detected to a similar extent in all the samples (Fig. 4c) while, IL-10 and IL-17 were below detection limits (data not shown). Cytokines were not detected in mock transfected DC stimulated with antigen alone (Fig. 4a–c). This indicates that TNF-α and IFN-γ secretion in T cell proliferation assay is primarily released from T cells.

We also examined IFN-γ and IL-4 levels in assays performed with CMV antigen which is known to induce Th1 polarization^24^. Similar to Ova, reduced secretion of IFN-γ was noted in miRNA mimic expressing DC compared to control Add- ‘while, IL-4 levels were below detection limit’. (Fig. 4d). Cytokine secretion was not detected in Ova pulsed DC (Fig. 4d). Taken together, these results show that Th1 activation-associated cytokine profiles are suppressed in DC transfected with miR-24, miR-30b, and miR-142-3p.

### miR-24, miR-30b, and miR-142-3p induce PD-L1 expression in APCs

**Figure 5:**
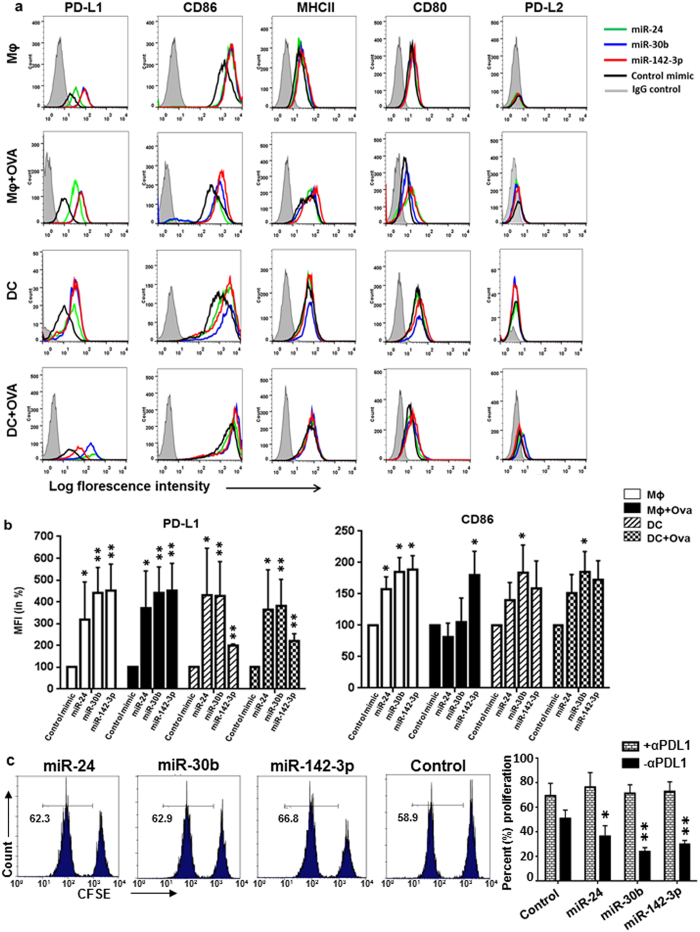
PD-L1 surface expression is induced in miR-24, miR-30b, and miR-142-3p transfected MΦ and DC. Cells were transfected with miRNA or control mimics and incubated with or without Ova. After 24 hrs, cells were harvested and stained for various surface markers. (**a**) Histograms showing expression of PD-L1, CD86, MHC-II, CD80, and PD-L2 as analyzed by flow cytometry. The gray shaded area represents isotype control. (**b**) Percent geometric MFI values for PD-L1 and CD86 in miRNA or control mimic transfected cells. (**c**) Antibody mediated blocking of PD-L1 restores inhibitory effect of miRNA induced PD-L1 expression on T cell proliferation. miRNA or control mimic transfected DC were incubated with PD-L1 antibody before co-culture with CFSE labelled T cells and CMV antigen. After 10 days, T cell proliferation was assessed by flow cytometry. Representative histograms and summary plots showing similar T cell proliferation in miRNA and control mimics transfected DCs. For comparison, T cell proliferation in miRNA mimic or control transfected DC without PD-L1 blocking is also presented. Data (n = 4) are representative of three independent experiments and are presented as mean ± SEM. Significance was determined by Student’s two-tailed *t*-test. *P < 0.05, **P < 0.01.

Antigen presentation is a highly regulated process and involves interaction of various molecules present on APC and T-cells. MHC-TCR interaction is the primary stimulus and must be facilitated by ligation of costimulatory molecules^25^. In this regard, CD80 and CD86 present on APC interact with CD28 on T-cells to trigger stimulatory signals, while PD-L1 and PD-L2 are ligands for programmed death-1 (PD-1) receptor on T-cells and negatively regulate T-cell priming^26^,^27^,^28^. We therefore examined the surface expression of MHC-II, CD80, CD86, PD-L1 and PD-L2 in MΦ and DC transfected with miRNAs. Marked induction (~2–4.5 fold) in PD-L1 expression was observed in miR-24, miR-30b, and miR-142-3p overexpressing cells compared to control mimic (Fig. 5a). On the other hand, increased CD86 expression was noticed but the fold induction was much smaller compared to PD-L1 (Fig. 5a,b). Enforced expression of miR-24, miR-30b, and miR-142-3p in untreated MΦ significantly induced (~1.5–2 fold) CD86 expression (Fig. 5a,b). However, CD86 expression was significantly elevated only in miR-142-3p transfected MΦ treated with Ova while Ova treated or untreated DC transfected with miR-30b showed significant changes (Fig. 5a,b). Surface levels of MHC-II and CD80 were not significantly impacted by miRNAs (Fig. 5a). We observed low PD-L2 expression in DC, but in MΦ it was below detection levels (Fig. 5a)^29^,^30^. However, PD-L2 levels in DC were not modulated by these miRs. Cells challenged with or without Ova exhibit similar impact on the expression of tested surface molecules indicating miRNA specific modulation.

Increased PD-L1 expression on APCs obstructs the proliferation of T cells. We next examined the impact of PD-L1 blocking on T cell proliferation by miR-24, miR-30b, and miR-142-3p. MiRNA or control mimic transfected DCs were stimulated with CMV antigen and PD-L1 was blocked by antibody before co-culture with autologous T cells. We observed similar T cell proliferation (~70–76%) in miRNA and control mimics (Fig. 5c). Moreover, we noticed higher T cell proliferation by blocking PD-L1 on DCs compared with experiments where PD-L1 was not blocked indicating a strong inhibitory role of PD-L1 on this process (Fig. 5c). Together, these results show that inhibitory impact of miRNA-mediated PD-L1 induction is effectively rescued by PDL-1 blocking.

## Discussion

In this study we demonstrate an inhibitory effect of miR-24, miR-30b, and miR-142-3p on the uptake as well as processing of Ova by APCs. Recently, we reported that overexpression of these miRNAs negatively impacts phagocytosis of bacteria and IgG opsonized beads and concomitant innate responses^20^. These results suggest a universal inhibitory role of these miRNAs in mediating particle internalization and activation of innate immune responses. Time kinetics of Ova uptake and processing reveals a similar impact of miR-24, −30b, and −142-3p on antigen uptake and processing by MΦ and DC at the early time points of 1.5 and 6 hr. However, at a later incubation time point (18 hr), we noted differential miRNA impact on antigen processing as observed by reduced antigen degradation in MΦ but not in DCs. These results suggest time dependent and cell specific regulation of antigen processing by miRNAs in MΦ and DC.

Uptake of Ova in DC is mediated by the mannose receptor (MR) or pinocytosis, while MΦ utilize scavenger receptors^31^. In DCs, MR-mediated uptake and pinocytosis respectively, leads to cross-presentation to CD8+ and CD4+ T-cells^32^. Evidently, MR deficient DC present antigen more efficiently to CD4+ T-cells, as this shifts the flux towards pinocytosis. In our previous study we observed reduced expression of both mannose receptor and scavenger receptors (MSR1 and MARCO) in miR-24, −30b or −142-3p overexpressing DC and MΦ^20^. Disruption of both antigen uptake pathways by miRNAs suggests that antigen presentation impacts not only CD4+ but also CD8+ T-cells to a similar extent. This is further supported by our two separate observations. Firstly, miR-24, −30b or −142-3p downregulate expression of multiple FcRs that plays important role in antigen uptake and presentation (Naqvi A.R, *In press*)^21^. Besides MHC-II presentation, antigen uptake by FcγRs allows APC to cross-present antigens to CD8^+^ T-cells^6^,^33^. The capability of FcRs to facilitate particle internalization and delivery to lysosomal compartments for degradation and antigen presentation is critical to their role in immunity. Consistent with this, lower FITC degradation was observed in cells overexpressing miRNAs after prolonged incubation (18 hours) indicating impaired antigen processing (Supplementary Figure 2). Secondly, we observed reduced Nox2 levels in miRNA-transfected MΦ and DCs upon incubation with opsonized beads (Naqvi A.R, *In press*)^21^. Antigen cross-presentation is closely associated with NADPH oxidase (Nox2) activity^34^,^35^,^36^. In DCs, Nox2 regulates antigen degradation efficiency and MHC-I cross-presentation to CD8+ T-cells. These observations illustrate miRα4-, −30b- or −142-3p-mediated defects in antigen processing and presentation by APCs and highlight their impact on downstream T-cell activation and effector functions.

APC-T-cell interaction is a prerequisite for productive antigen presentation and is highly dependent on cell mobility. Cell migration defects associated with miR-24, miR-30b and miR-142-3p are reported through targeting of key genes in cell cytoskeleton homeostasis^37^,^38^,^39^,^40^,^41^. Consistent with this, our previous results show that these miRNAs impact expression of various genes associated with phagocytosis^20^. Many of these regulate cellular structural dynamics, including Phospholipase C (PLC) and Protein Kinase C (PKC) family members. These results lend support to our hypothesis that in myeloid inflammatory cells, miR-24, −30b and −142-3p predominantly regulate critical components of cytoskeleton dynamics leading to altered cell morphology resulting in significant impairment of their capacity to efficiently process and present antigen to T-cells.

Imbalance in Th1 and Th2 cytokines is responsible for a variety of immunoinflammatory disorders including multiple sclerosis, arthritis, asthma, cancer, etc.,^42^,^43^. Emerging lines of evidences highlight the regulatory roles for miRNA in Th polarization. For instance, ablation of miR-21 in mice significantly increased levels of the Th1 cytokine IFN-γ. Upon challenge with LPS, DCs from miR-21^−/−^ mice produce less IL-12p35, a key cytokine that drives IFN-γ production^17^. Similarly, Th2 polarization is regulated by T-cell intrinsic miR-155, while miR-146a controls Th1/Th17 skewing^43^,^44^. Our results show that CD4+ T-cells co-cultured with APCs overexpressing miR-24, −30b or −142-3p mimics are less efficient in secreting IFN-γ and TNF-α cytokines in the presence of Ova as well as reduced IFN-γ levels in assays performed with Th1 inducing antigen derived from CMV. These results indicate that miRNA-mediated defects in APC function could interfere with T-cell effector functions. MiRNA-mediated defects in Th1 polarization are also supported by our earlier findings that these miRNAs downregulated IL-12p35 secretion by MΦ and DC challenged with *E. coli* or purified LPS^19^,^20^. Expression of miR-142-3p reduces during M1 and M2 differentiation; however anti-inflammatory M2 MΦ were found to have lower miR-142-3p expression^19^,^20^,^45^. Taken together these findings suggest that miRNA expression regulates APC polarization as well as adaptive immune cell polarization by modulating secretion of cytokines that may play a crucial role in Th polarization.

Our results show induced PD-L1 expression in miR-142-3p-transfected cells. High levels of miR-142-3p were reported during myeloid cell differentiation in leukemia cell lines and CD34(+) hematopoietic stem/progenitor cells^46^,^47^,^48^. Similarly, miR-142-3p was upregulated in human T-leukemic cell lines and primary T-leukemic cells isolated from T-cell acute lymphoblastic leukemia (T-ALL) patients and its expression levels correlated with prognosis^49^. Interestingly, increased expression of PD-L1 has been reported in chronic lymphocytic leukemia (CLL) suggesting a possible association of miR-142-3p and PD-L1 expression^50^. Increased PD-L1 prevents efficient activation and proliferation of T-cells allowing immune evasion by pathogen and tumors^51^. Conversely, blocking PD-1:PD-L1 pathway using monoclonal antibodies can reverse tumor immune evasion leading to robust antitumor responses^52^,^53^,^54^,^55^. Our results confirm that PD-L1 blocking relieves miRNA-mediated inhibition of T cell priming by DCs. In MΦ, *Mycobacteria tuberculosis* (M. tb) infection induces high levels of miR-142-3p and impairs phagocytosis of pathogen^56^. In our previous study we showed that enforced expression of miR-142-3p in myeloid inflammatory cells results in defective phagocytosis as well as reduced secretion of proinflammatory cytokines^20^. These findings indicate that aberrant miR-142-3p and PD-L1 levels can suppress both innate and adaptive immune responses. Overall, our results highlight novel mechanistic insights through which miR-24, miR-30b and miR-142-3p can regulate activation of adaptive immune responses guided by APCs.

## Methods

### Study Approval

All mouse procedures were approved by the Institutional Animal Care and Use Committee of the University of Illinois at Chicago (ACC 15-003) and all experiments were performed in accordance with the appropriate guidelines and regulations.

### Mice

C57BL/6 (B6) and mice were obtained from The Jackson Laboratory (Bar Harbor, ME). Mice were housed under specific-pathogen–free conditions. All experiments were conducted on 8- to 12-week-old mice.

### Primary human monocyte isolation and differentiation

Freshly prepared buffy coats were collected from healthy donors (Sylvan N. Goldman Oklahoma Blood Institute, Oklahoma City, OK, USA) by density gradient centrifugation as described previously^19^,^20^. Briefly, PMBCs were purified using Ficoll Paque^TM^ (Fisher Scientific, Pittsburgh, PA, USA) based density centrifugation. PBMCs were incubated with magnetic labeled CD14 beads (Miltenyi Biotech, San Diego, CA, USA) according to manufacturer’s instructions. The purity of CD14+ cells was >95% as determined by flow cytometry. For MΦ differentiation, monocytes were plated at 2 × 10^6^/ml in DMEM supplemented with penicillin (100 U/ml) and streptomycin (100 μg/ml). After 2 h the media was substituted with media containing 10% FBS (Life Technologies, Carlsbad, CA, USA), and rhM-CSF (50 ng/mL; Peprotech, Rocky Hill, NJ, USA). For DCs, monocytes were cultured in RPMI-1640 supplemented with rhGM-CSF (1000 U/ml) and rhIL-4 (500 U/ml) (both from Peprotech). Media was replaced every 48 h. At day 7, cells were harvested and differentiation confirmed by flow cytometric analysis as described earlier^19^,^20^.

### Differentiation of murine bone marrow derived MΦ and DC

Bone marrow was isolated from 8–12 week old mice. Murine bone marrow derived DC (BMDC) were cultured in murine recombinant rmuGM-CSF (1000 U/ml) and rmuIL-4 (500 U/ml) (all from Peprotech), respectively. Flow cytometry analysis show that day 7 differentiated BMDC were >65% CD11c positive (Supplementary Figure 3a).

### Transient miRNA transfections

MiR-24, miR-30b, and miR-142-3p mimics or inhibitors were purchased from Qiagen (Gaithersburg, MD, USA). As a negative control, miRNA mimics with no known human targets was used (Qiagen). Transient transfection of miRNA mimics, inhibitors or control was performed using Lipofectamine 2000 (Invitrogen, Carlsbad, CA). MΦ were transfected at a final concentration of 50 nM oligos while DC were transfected with 100 nM oligos. As a positive transfection control, we used DY-547 labelled siRNA (Life Technologies).

### Antigen uptake and processing by APCs

MiRNA mimic or control transfected DC, MΦ, BMDC were challenged with Texas Red- or DQ^TM^-conjugated Ova (1 mg/ml, Molecular Probes, Grand Island, NY) in respective complete media for various time points at 37 °C. DQ^TM^-Ova consist of Ova that are heavily conjugated with BODIPY FL, resulting in self-quenching. Upon proteolytic degradation of DQ-Ova to single dye-labeled peptides, bright green fluorescence is observed. After assay incubation, cells were washed thrice with PBS, fixed with 2% PFA and mounted in Prolong Gold Antifade with DAPI (Life Technologies). For FACS, cells were harvested with Accutase (Cell Biolabs, San Diego, CA) treatment, washed twice with 1X PBS/0.1% BSA and analyzed on BD Fortessa (BD Biosciences, Franklin Lakes, NJ). Cells were imaged on LSM 710 Meta confocal microscope for antigen processing as described below. Cells were also harvested and analyzed on BD Gallios using fluorophore specific settings. The ratio of geometric mean florescence intensity (geo. MFI) value of positive control and miRNA mimics, inhibitor or control mimic was obtained. These ratios were converted to percentages. Antigen uptake and processing was monitored by measuring Texas Red and BODIPY florescence, respectively.

### Confocal imaging

Images were captured on Zeiss LSM 710 confocal microscope with 40×/1.2 Water DIC C-Apochromat objective (ZEISS Microscopy, Oberkochen, Germany). Confocal images were processed on ZEN lite software (ZEISS Microscopy). Images from five randomly selected fields were captured for each donor (n = 4).

### ELISA

Supernatants levels of TNF-α, IL-4, IL-10, IL-17 and IFN-γ were measured by ELISA (Cytoset, Invitrogen). Supernatants were diluted (1:5) before performing the assay. The absorbance was measured at 450 nm using the SpectraMax^®^ M2 (Molecular Devices, Sunnyvale, CA).

### Flow cytometry

Cells were harvested after treatments, washed with ice cold PBS and fixed with 2% paraformaldehyde for 15 mins. After washing, cells were resuspended in 50–100 μL of FcR blocking reagent (Miltenyi Biotech), followed by 15 min incubation at room temperature (RT) to allow blocking of Fc receptors. The cell pellet was washed twice, resuspended in 50 μL 2% BSA/TBS (w/v), and incubated with fluorochrome-conjugated antibodies (1:50 or 1:100 dilution). The following antibodies were used: Human CD4, CD80, CD86, MHC-I, MHC-II, PD-L1 and PD-L2 (BD Pharmingen), mouse CD4, CD11c, F4/80 (eBiosciences, San Jose, USA). Samples were analyzed using a FACScan or BD Cyan flow cytometer using CellQuest software (BD Biosciences). Further analysis was performed using FlowJo software (Tree Star Inc.).

### T-cell activation and proliferation assays

Day 7 differentiated MΦ were plated at 3 × 10^4^ per well while DCs were seeded at a density of 2 × 10^4^ in 96-well round bottom plates. Cells were transfected with miRNA mimics and the following day stimulated overnight with Ova (10 μg/ml; grade V, Sigma Aldrich, St. Louis, MO), or CMV antigen (20 μg/ml; Meridian Life Sciences, Memphis, TN). CD4+ T-cells were isolated by negative selection using magnetic separation as per manufacturer’s instructions (Miltenyi). In our hands, the purity of these cells populations was >95% (Supplementary Figure 3b). Isolated T-cells were labeled with CFSE (2 μM; Molecular Probes) and co-cultured with APCs at 5:1 ratio for 10 days. At the end of assay, T-cells were analyzed on a Fortessa cytometer (BD Biosciences) and data was further analyzed using Kaluza software (BD Biosciences). Cell proliferation assays were also performed by blocking PD-L1 using PD-L1 antibodies. MiRNA or control mimic transfected DCs were stimulated with CMV antigen for overnight and incubated with 4.0 μl PD-L1 antibodies (1:50 dilution; clone MIH1; BD Pharmingen) and CFSE labelled T cells. PD-L1 antibodies were added during media replacement. For T-cell proliferation assays using OT-II mice derived BMDCs, CD4+ T-cells were sorted from total splenic population of naive transgenic mice (OT-II). BMDCs (2 × 10^4^) transfected with murine miRNAs were co-cultured with CFSE labeled CD4+ T-cells in the presence of Ova (triplicate cultures). After 4 days, cells were harvested and analyzed on flow cytometer. Percentages of CD4+ CFSE+ gated T-cells were determined using Kaluza software (BD Biosciences).

### Statistical Analysis

Data were analyzed on GraphPad Prism (La Jolla, USA). The results are represented as standard deviation or ±SEM from three independent replicates and experiments were conducted at least thrice. P-values were calculated using Students *t*-test and P < 0.05 were considered significant. *P  <  0.05, **P  <  0.01, ***P  <  0.001. For time kinetics analysis, we used identical time points to compare differences due to miRNA and control mimic transfection on antigen processing and uptake. Because these processes increase over time as observed with increase in both BODIPY and Texas Red intensity, we avoided comparison across different time points and therefore the significance values were derived from t-test analysis.

## Additional Information

**How to cite this article**: Naqvi, A. R. *et al*. miR-24, miR-30b and miR-142-3p interfere with antigen processing and presentation by primary macrophages and dendritic cells. *Sci. Rep.* **6**, 32925; doi: 10.1038/srep32925 (2016).

## Supplementary Material

Supplementary Information
