# Supplementary material for: miR-24, miR-30b and miR-142-3p interfere with antigen processing and presentation by primary macrophages and dendritic cells
Source: Sci Rep. 2016 Sep 9;6:32925. doi: 10.1038/srep32925 (PMC5017188; doi:10.1038/srep32925)
Supplement: Supplementary Information [file srep32925-s1.pdf]

Afsar Raza Naqvi, Jezrom B. Fordham, Balaji Ganesh, Salvador Nares. miR-24, miR-30b and miR-142-3p interfere with antigen processing and presentation by primary macrophages and dendritic cells.

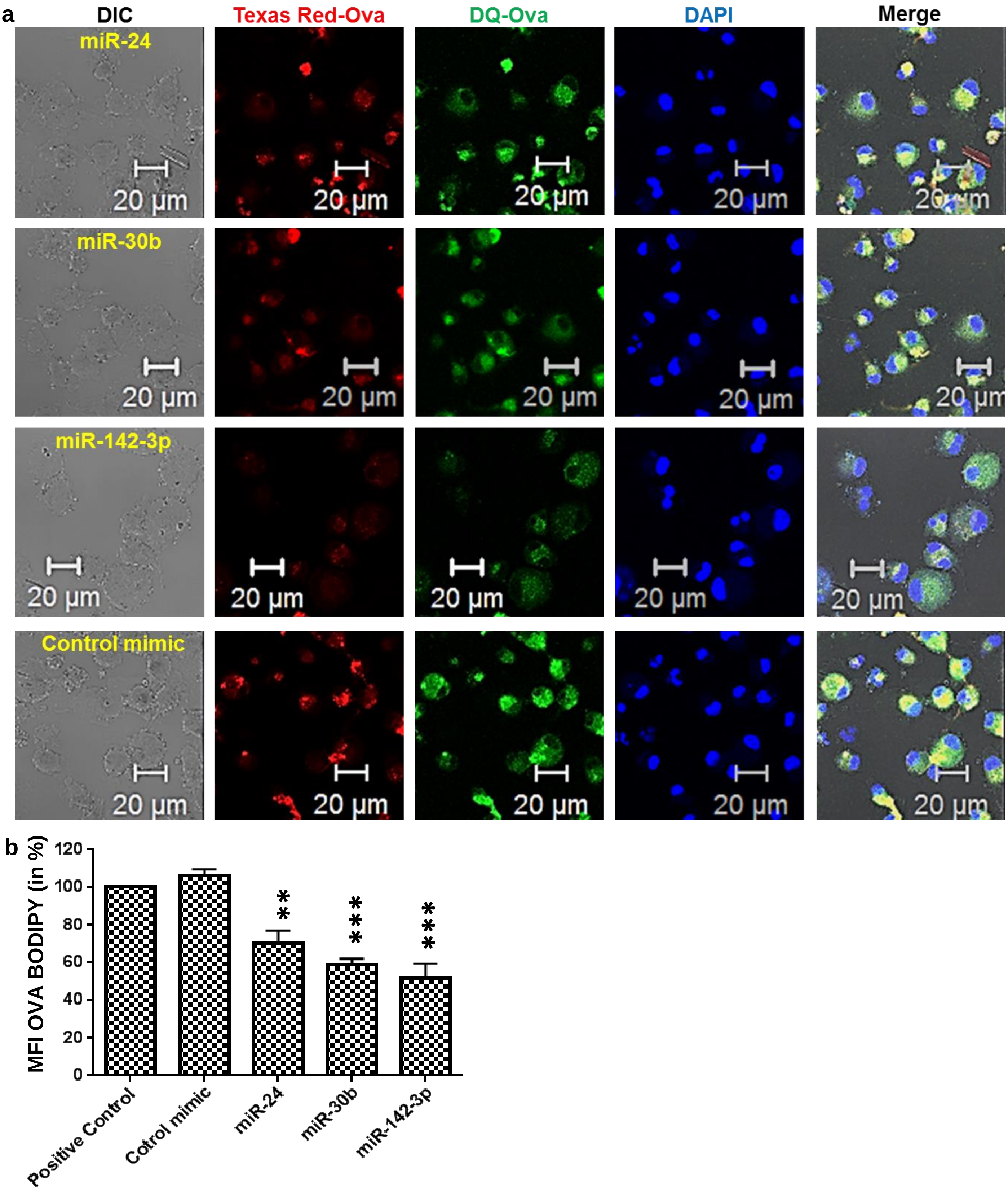

Supplementary Figure 1. miR-24, miR-30b and miR-142-3p attenuate antigen uptake and processing in murine BMDC. Cells were transfected with murine miRNA or control mimics and assayed for their impact on uptake and processing of antigens by using Ova-Texas Red and Ova-BODIPY, respectively. (a) Representative images showing miRNA-24, -30b and -142-3p mediated reduction of Ova uptake and processing in BMDCs. Scale bar, 20 μm. (b) Histograms showing geometric MFI values (in %) of processed Ova-BODIPY in BMDCs transfected with miRNA and control mimics. Data are representative of three independent experiments (n=4) and are presented as mean ± SEM. Significance was determined by Student's two-tailed t-test. **P< 0.01, ***P<0.001.

Afsar Raza Naqvi, Jezrom B. Fordham, Balaji Ganesh, Salvador Nares. miR-24, miR-30b and miR-142-3p interfere with antigen processing and presentation by primary macrophages and dendritic cells.

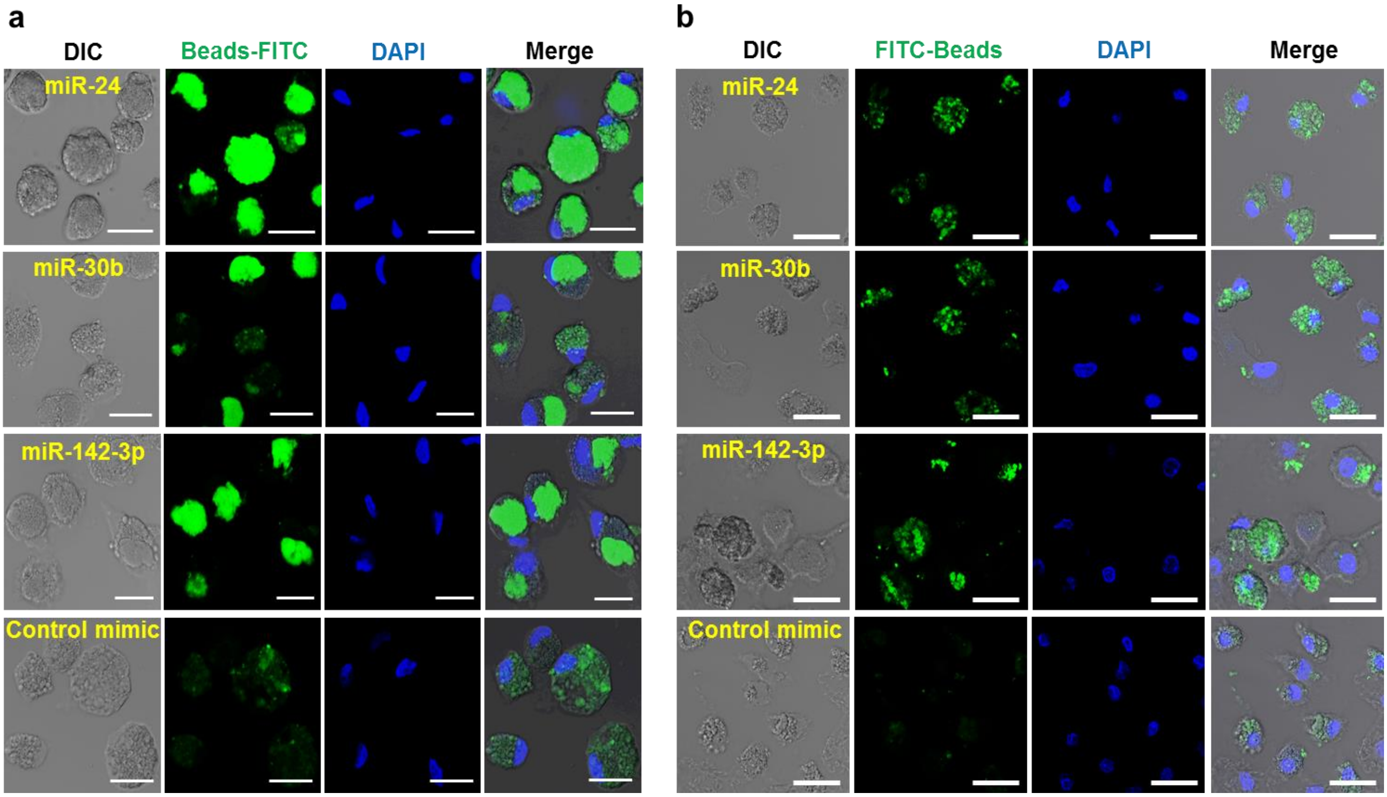

Supplementary Figure 2. Delayed degradation of internalized antigen in miR-24, miR-30b and miR-142-3p transfected MΦ and DC. MiRNA mimic or control transfected cells were incubated with IgG opsonized FITC conjugated beads. Cells were incubated overnight and images were captured by confocal microscopy. Representative images showing defective degradation of antigen (FITC) beads in miR-24, miR-30 and miR-142-3p transfected (a) MΦ and (b) DC compared to control mimic. Scale bar, 20 µm. Images were captured from three different fields of the same well. Experiments were conducted on three independent donors.

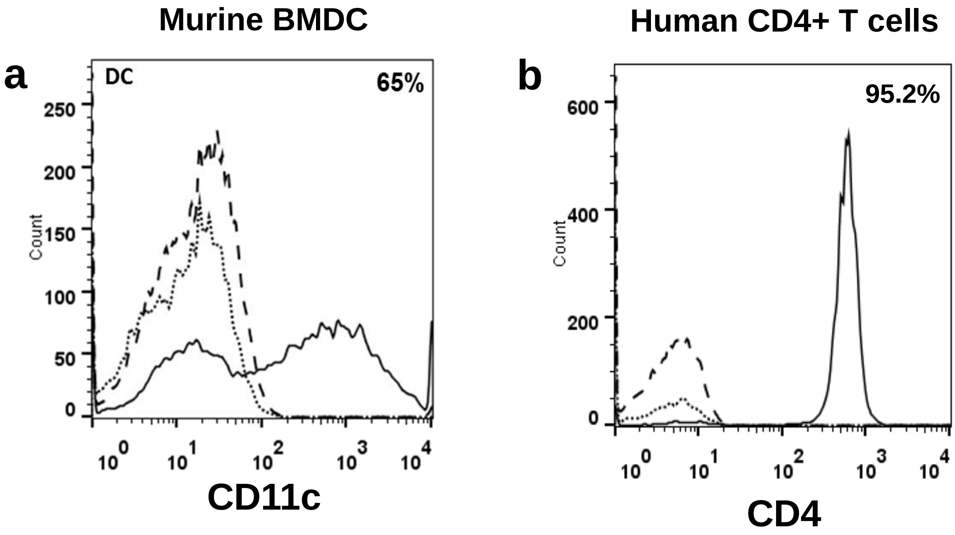

Supplementary Figure 3. Flow analysis of murine BMDC and human T-cell surface markers. (a) Histogram showing CD11c surface expression in day 7 differentiated murine BMDC analyzed by flow cytometry. (b) Human T-cells were isolated from independent donors by negative selection. The purity of CD4+ T-cells population was verified by flow cytometry.
